# Identification of endophenotypes supporting outcome prediction in hemodialysis patients based on mechanistic markers of statin treatment

**DOI:** 10.1016/j.heliyon.2024.e30709

**Published:** 2024-05-06

**Authors:** Johannes Leierer, Madonna Salib, Michail Evgeniou, Patrick Rossignol, Ziad A. Massy, Klaus Kratochwill, Gert Mayer, Bengt Fellström, Nicolas Girerd, Faiez Zannad, Paul Perco

**Affiliations:** a Medical University of Innsbruck, Department of Internal Medicine IV, Innsbruck, Austria; b Université de Lorraine, Inserm, Centre d’Investigations Cliniques- 1433, and Inserm U1116, CHRU Nancy, F-CRIN INI-CRCT, Nancy, France; c Medical University of Vienna, Comprehensive Center for Pediatrics, Department of Pediatrics and Adolescent Medicine, Division of Pediatric Nephrology and Gastroenterology, Vienna, Austria; d Medical Specialties and Nephrology departments, Princess Grace Hospital, Monaco, Monaco; e Association pour l'Utilisation du Rein Artificiel (AURA) Paris and Department of Nephrology, CHU Ambroise Paré, APHP, 92104, Boulogne Billancourt, and Centre for Research in Epidemiology and Population Health (CESP), University Paris-Saclay, University Versailles-Saint Quentin, Inserm UMRS, 1018, Clinical Epidemiology Team, Villejuif, France; f Uppsala University, Department of Medical Sciences, Uppsala, Sweden

**Keywords:** Statins, Gene expression profiling, Predictive biomarkers, Patient stratification, Cox proportional hazards regression models, Cardiovascular risk, Hemodialysis, AURORA cohort

## Abstract

**Background:**

Statins are widely used to reduce the risk of cardiovascular disease (CVD). Patients with end-stage renal disease (ESRD) on hemodialysis have significantly increased risk of developing CVD. Statin treatment in these patients however did not show a statistically significant benefit in large trials on a patient cohort level.

**Methods:**

We generated gene expression profiles for statins to investigate the impact on cellular programs in human renal proximal tubular cells and mesangial cells in-vitro. We subsequently selected biomarkers from key statin-affected molecular pathways and assessed these biomarkers in plasma samples from the AURORA cohort, a double-blind, randomized, multi-center study of patients on hemodialysis or hemofiltration that have been treated with rosuvastatin. Patient clusters (phenotypes) were created based on the identified biomarkers using Latent Class Model clustering and the associations with outcome for the generated phenotypes were assessed using Cox proportional hazards regression models. The multivariable models were adjusted for clinical and biological covariates based on previously published data in AURORA.

**Results:**

The impact of statin treatment on mesangial cells was larger as compared with tubular cells with a large overlap of differentially expressed genes identified for atorvastatin and rosuvastatin indicating a predominant drug class effect. Affected molecular pathways included TGFB-, TNF-, and MAPK-signaling and focal adhesion among others. Four patient clusters were identified based on the baseline plasma concentrations of the eight biomarkers. Phenotype 1 was characterized by low to medium levels of the hepatocyte growth factor (HGF) and high levels of interleukin 6 (IL6) or matrix metalloproteinase 2 (MMP2) and it was significantly associated with outcome showing increased risk of developing major adverse cardiovascular events (MACE) or cardiovascular death. Phenotype 2 had high HGF but low Fas cell surface death receptor (FAS) levels and it was associated with significantly better outcome at 1 year.

**Conclusions:**

In this translational study, we identified patient subgroups based on mechanistic markers of statin therapy that are associated with disease outcome in patients on hemodialysis.

## 1 Background

Statins are among the most prescribed drugs in the world. They exert their lipid-lowering effect by targeting the 3-hydroxy-3-methylglutaryl-CoA reductase (HMGCR), better known as HMG-CoA reductase, one of the key enzymes in the cholesterol synthesis pathway. Statin use is recommended for adults at risk of developing cardiovascular disease (CVD) [1,2]. Variability in response to statin therapy is however substantial and there are currently no good parameters for defining statin responders. Next to this variability in response, considerable side effects seem to be associated with statin therapy such as an elevated risk of diabetes mellitus, elevated transaminase levels, myalgia, or rhabdomyolysis [[3], [4], [5]]. In addition, some interfere with the metabolism of other drugs making combination therapy complex.

Patients with end-stage renal disease (ESRD) on hemodialysis have a significantly increased risk of developing CVD. Large trials such as AURORA or the German 4D study investigating the impact of rosuvastatin and atorvastatin on clinical outcomes in hemodialysis patients however failed to demonstrate a beneficial impact of statin treatment in this patient population on the cohort level [6,7].

Researchers have therefore started to search for predictive markers for statin therapy to identify responders and optimize therapeutic use of statins. These approaches included pharmacogenomic markers [8] as also reviewed by Arrigoni and colleagues [9], pharmacometabolomic markers [10], and recently also in linking changes in the human gut microbiome with response to statin therapy [11]. Associations between gene polymorphisms in HMGCR and concentrations of inflammatory serum markers that might influence the response to statin therapy regarding the anti-inflammatory potential have been recently described [12]. Other groups focused on clinical parameters in calculating risk scores to guide therapeutic intervention in hemodialysis patients [13].

In this study we investigated the effect of atorvastatin and rosuvastatin on the transcriptional level in renal mesangial and proximal tubular cells using whole genome microarrays. We analyzed changes in gene expression on the level of molecular pathways and selected a panel of biomarkers for measurements in plasma samples from the AURORA study, a double-blind, randomized, multi-center study of patients on hemodialysis or hemofiltration. The marker levels were used to stratify patients, and resulting phenotypes were evaluated regarding their association with disease outcome and response to statin therapy.

## 2 Methods

### 2.1 Renal cell culture models

Normal human mesangial cells (MCs) were purchased from Lonza (CC-2559, Basel, Switzerland) and cultured according to manufacturer's protocol in MsGM medium (Lonza, CC-3147). Proximal tubular human kidney cells (HK2) were purchased from American Type Culture Collection (CRL-2190, Wesel, Germany) and cultured in Keratinocyte-Serum Free Medium (KSFM) containing 10 % fetal bovine serum (FBS), 5 ng/ml recombinant epidermal growth factor (rEGF), 0.05 mg/ml bovine pituitary extract (BPE), 100 U/ml penicillin and 100 μg/ml streptomycin. All cells were grown at 37 °C in a humidified atmosphere with 5 % CO_2_. After growth to confluency, cells were serum deprived and stimulated with high glucose (30 mM) for 24 h. Thereafter cells were treated with 20 μM atorvastatin or rosuvastatin for 24 h. Total RNA was isolated with RNeasy Mini Kit (Qiagen, Valencia, CA, USA) according to the manufacturer's protocol. RNA yield and quality was determined using a DS-11 FX + spectrophotometer (DeNovix, Wilmington, DEL, USA).

### 2.2 Transcriptomics profiling and gene expression analysis

All materials for microarray experiments were purchased from Agilent (Agilent Technologies, Inc, USA) unless otherwise specified. For each sample 200 ng total RNA was used. Cyanine 3 (Cy3) labelled cRNA was generated with the LowInput QuickAmp Labeling Kit (5190-2305) for hybridization to oligonucleotide microarrays (Human GE 4 × 44K v2, G4845A). Arrays were scanned at 5 μm resolution using an Axon Gene Pix 4000B scanner (Molecular Devices, Sunnyvale, CA) and signal intensities were extracted using Agilent Feature Extraction software (v.9.5.3.1).

Microarray preprocessing and statistical analysis was conducted with the limma package of the statistical software framework R [14]. Arrays of HK2 and MCs were preprocessed separately. Preprocessing consisted of background correction, quantile normalization as well as summarization of probes with the same sequence spotted multiple times on the array. The moderated t-statistics with adjustment for multiple testing setting the false discovery rate (FDR) to < 5 % was used to identify differentially expressed transcripts between cells treated with either atorvastatin or rosuvastatin and untreated cells respectively. The overlap between sets of identified differentially expressed transcripts was visualized with a Venn diagram. Differentially expressed transcripts were further annotated with Ensembl GeneIDs via Ensembl Biomart and the number of unique protein coding differentially expressed genes (DEGs) was determined.

Microarray data are deposited in the Gene Expression Omnibus with accession number GSE208579.

### 2.3 Pathway enrichment analysis and biomarker selection

The Database for Annotation, Visualization and Integrated Discovery (DAVID) v6.8 was used for functional enrichment analysis [15]. The sets of DEGs for the two drugs and the two cell lines were separately used as input for identification of enriched molecular pathways making use of the KEGG pathway database [16]. Pathways with enrichment p-values smaller than 0.1 were considered as relevant. Relevant pathways were ranked in ascending order based on the sum of the four individual p-values.

Biomarkers for measurements in the AURORA study were selected based on their regulation by statin treatment in-vitro, their involvement in mechanistic processes linked to statin treatment, their associations to CVD and CKD, as well as based on the availability of analytes on the respective OLink panels.

### 2.4 Biomarker measurements in the AURORA study

Biomarkers were measured in 398 baseline samples of the AURORA (A study to evaluate the Use of Rosuvastatin in subjects On Regular hemodialysis: an Assessment of survival and cardiovascular events) trial, a double-blind, randomized, multi-center study of patients on hemodialysis or hemofiltration for at least three months. After providing written informed consent, eligible patients were randomly assigned in a 1:1 ratio to the treatment arm (10 mg daily dose of rosuvastatin) or the placebo group. Description, baseline data and main results of the AURORA study have been published previously [6,17,18]. In brief, rosuvastatin did not show a significant beneficial impact in the overall cohort with respect to the composite primary endpoint of MACE nor the secondary endpoint of all-cause mortality.

The selected biomarkers were measured with the Olink Proseek Multiplex CVD panels II and III as well as the Inflammation panel in plasma samples (Olink Proteomics AB, Uppsala, Sweden). The platform is based on a proximity extension assay (PEA) technology where 92 oligonucleotide-labelled antibody probe pairs per panel are allowed to bind to their respective targets in 1 μL plasma in a 96-well plate format. When binding to their correct targets, they give rise to new DNA amplicons each ID-barcoding their respective antigens. The amplicons are subsequently quantified using a Fluidigm BioMark HD realtime polymerase chain reaction (PCR) platform. The platform provides log2 Normalized Protein eXpression (NPX) data wherein high protein values correspond to high protein concentrations, but not an absolute quantification (https://olink.com/faq/what-is-npx/).

### 2.5 Patient stratification based on molecular biomarkers in the AURORA study

We computed residuals from fitted linear regression models, where each protein biomarker was used as dependent variable and sex, age, and calculated Kt/V were used as independent variables. Latent Class Model clustering was performed on the residuals to identify patient patterns using the VarSelLCM R package.

To assess the biomarker relevance for each phenotype, we generated a decision tree to predict the phenotypes based on biomarker concentrations using the rpart R package. The minimum number of observations to be present in a node in order to perform additional splits was set to be >10 % of the total patient population.

### 2.6 Statistical outcome analysis

Comparisons of patient characteristics across the identified phenotypes were analyzed using analysis of variance (ANOVA), Kruskal-Wallis-, and chi-squared tests, as appropriate. Categorical variables are expressed as frequencies (percentages), whereas continuous variables are expressed as means ± standard deviation (SD) for normally distributed data and median along with interquartile ranges (25th and 75th percentiles) for non-normally distributed data.

The associations between phenotypes and outcome (CV death, all-cause mortality, and MACE) were assessed using Cox proportional hazards regression models. The multivariable models were adjusted for clinical and biological covariates based on previously published data in AURORA (i.e. age, history of CV disease, diabetes mellitus, serum albumin, high sensitivity C-reactive protein (hs-CRP), dialysis vintage, and B-type natriuretic peptide (BNP)) [19]. History of CV disease is a composite variable of history of coronary heart disease (i.e. prior myocardial infarction, prior coronary angioplasty or stent, and coronary artery bypass graft), history of vascular disease (i.e. peripheral artery disease, abdominal aortic aneurysm, carotid artery disease, carotid stenosis ≥ 50 %, and carotid endarterectomy), and history of neurovascular disease (i.e. prior ischemic vascular accident and transient ischemic attack). BNP and hs-CRP were best modelled by using their natural logarithm.

The Kaplan Meier method was used to estimate risk for each outcome according to phenotypes and are presented as event curves. The risk differences at one year, two years, and three years with conﬁdence intervals (CI) at 95 % between rosuvastatin and placebo groups are provided.

All statistical analyses were performed using R version 4.0.2 software (R Development Core Team).

## 3 Results

Statin treatment significantly impacts gene expression in proximal tubular cells and mesangial cells in-vitro.

**Fig. 1:**
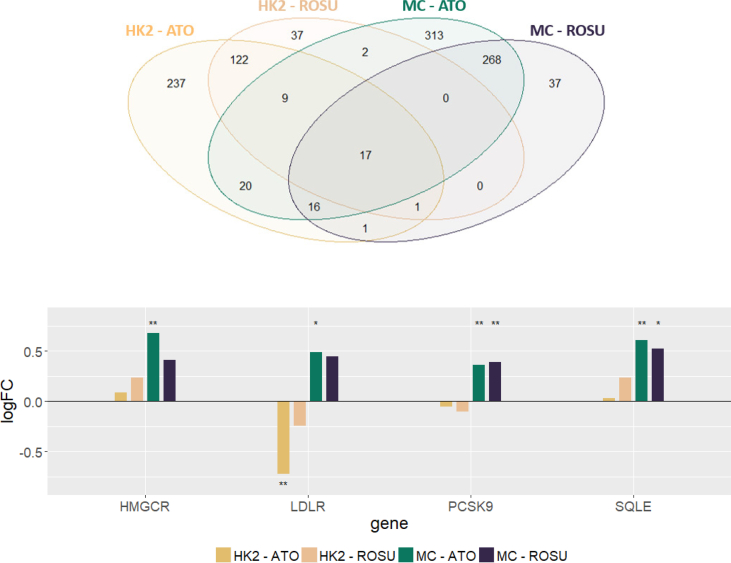
Statin treatment effect on gene expression in renal cells *Panel A:* The number of differentially expressed transcripts between treated and untreated cells for the two cell lines studied is depicted in the Venn diagram. A set of 17 transcripts was deregulated in both cell lines by both drugs studied. *Panel B:* Graphical representation of log fold-change (logFC) values of the direct statin drug target HMGCR and associated genes LDLR, PCSK9, and SQLE. Significant fold-change values are indicated with ** (FDR <5 %) and * (FDR <10 %) respectively.

34,127 unique transcripts remained after background correction, quantile normalization and probe summarization for statistical analysis. Rosuvastatin treatment significantly affected 188 transcripts in HK2 cells and 340 transcripts in MCs which could be mapped to 167 and 304 unique protein coding genes respectively (see Fig. 1A). Atorvastatin treatment had a larger impact on gene expression changes than rosuvastatin, significantly affecting gene expression of 423 transcripts in HK2 cells and 646 transcripts in MCs. These transcripts could be mapped to 388 and 570 unique protein coding genes respectively.

Significant overlaps between DEGs affected by both drugs could be detected in HK2 cells (p-value <0.0001) as well as in MCs (p-value <0.0001) based on chi-squared tests. This set of molecules affected by both drugs represents the drug class effect, and of note that not a single feature in this set was upregulated by one drug and downregulated by the other drug thus further strengthening the quality of the generated gene expression dataset.

The overall effect on gene expression changes was larger in MCs as compared to HK2 cells with in total 684 differentially expressed transcripts in MCs as compared to 462 differentially expressed transcripts in HK2 cells.

A set of 17 genes was affected by both drugs in both cell lines. 13 were upregulated after statin treatment including for example the pro-fibrotic connective tissue growth factor (CTGF). Three genes were downregulated after statin treatment including for example the Kruppel like factor 2 (KLF2). One gene, namely the zinc finger BED-type containing 2 (ZBED2), was upregulated in MCs but downregulated in HK2 cells.

Statin treatment affects gene expression of its direct target an associated genes linked to cholesterol biosynthesis.

We observed a significant upregulation of HMGCR on the mRNA level in MCs but not in HK2 cells (see Fig. 1B). In addition to HMGCR, also the receptor for low density lipoprotein (LDLR) was upregulated in MCs but not in HK2 cells in our study. The proprotein convertase subtilisin/kexin type 9 (PCSK9) and squalene epoxidase (SQLE), one of the rate-limiting enzymes in the sterol biosynthesis pathway was also significantly upregulated in MCs after statin treatment in our experiments.

Next to the direct target of statins and additional members of the cholesterol biosynthesis we focused on selected key processes being enriched in DEGs in order to investigate the pleiotropic effects of statins on renal cells.

Statin treatment significantly impacts molecular pathways and molecular biomarkers linked to CKD and CVD progression.

29 molecular KEGG pathways were identified as being enriched based on at least one of the four DEG lists.

The top ranked enriched molecular KEGG pathway based on the sum of p-values of the four comparisons was the MAPK signaling pathway. Among the top enriched pathways were also the fibrosis related TGF-beta signaling pathway, the inflammation related pathways TNF-signaling and leukocyte transendothelial migration but also pathways on focal adhesion and mineral absorption (see Table 1).

**Table 1:**
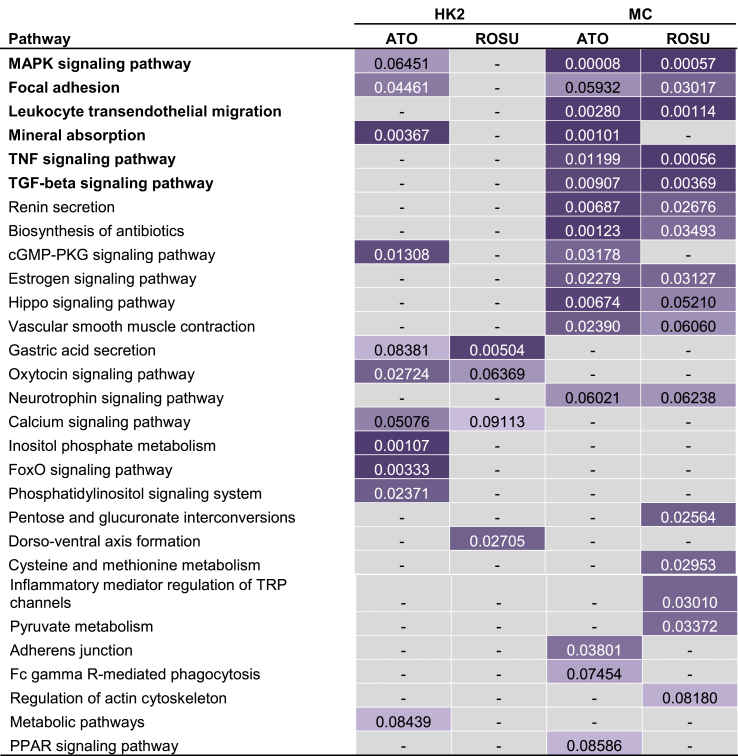
Affected molecular pathways after statin therapy.

Eleven genes linked to the fibrosis related TGF-beta signaling pathway were identified as differentially expressed after statin treatment, mainly in MCs. The set of downregulated genes contained the two TGF-beta isoforms 2 (TGFB2) and 3 (TGFB3) as well as the growth differentiation factor 6 (GDF6), bone morphogenetic protein 4 (BMP4) as well as follistatin (FST). One of the endogenous inhibitors of the TGF-beta signaling pathway, SMAD6, on the other hand, was induced by statin treatment in-vitro. The impact of atorvastatin and rosuvastatin on gene expression of TGF-beta associated transcripts was very consistent indicating a drug class effect on this molecular process.

A clear difference in the regulation of MAPK components is apparent in the two cell types under study. Whereas a large number of molecules from the MAPK signaling pathway is upregulated in HK2 cells with almost none of the MAPK signaling members being downregulated, there are twice as many downregulated than upregulated MAPK signaling molecules in MCs.

Factors being associated with leukocyte transendothelial migration that were upregulated by statins in our cell lines included among others CXCL12, CLDN4, Platelet F11 receptor (F11R), or MMP2. Components of the TNF signaling pathway were mainly downregulated by statins in MCs in our experiment. This downregulation could be observed on the level of intracellular signaling molecules such as MAP2K3 but also more importantly on the effector level of this signaling pathway. The inflammatory molecules IL6, CCL20, CSF2, and ICAM1 for example were significantly downregulated after statin treatment. Also endothelin 1 (EDN1) was markedly downregulated by both statins in MCs.

Another sign of evidence for the impact on cell proliferation and cell differentiation of statins is the fact that a number of components of cell-matrix adhesion proteins is dysregulated as compared to unstimulated cells. Various actinin isoforms (ACTN1, ACTN3, ACTN4) were found to be downregulated after statin treatment next to downregulation of caveolin 1 (CAV1) and 3 (CAV3), linking integrins to intracellular signaling pathways thus promoting cell cycle progression. In addition, filamin (FLNA), vinculin (VCL) and zyxin (ZYX) were found to be downregulated after statin treatment. Extracellular molecules like integrin beta 4 (ITGB4), integrin alpha 7 (ITGA7) or the hepatocyte growth factor (HGF) on the other hand were significantly upregulated by statins.

The expression of another major renoprotective factor, namely heme oxygenase 1 (HMOX1), was significantly induced by atorvastatin but also upregulated by rosuvastatin in both cell types under study.

**Fig. 2:**
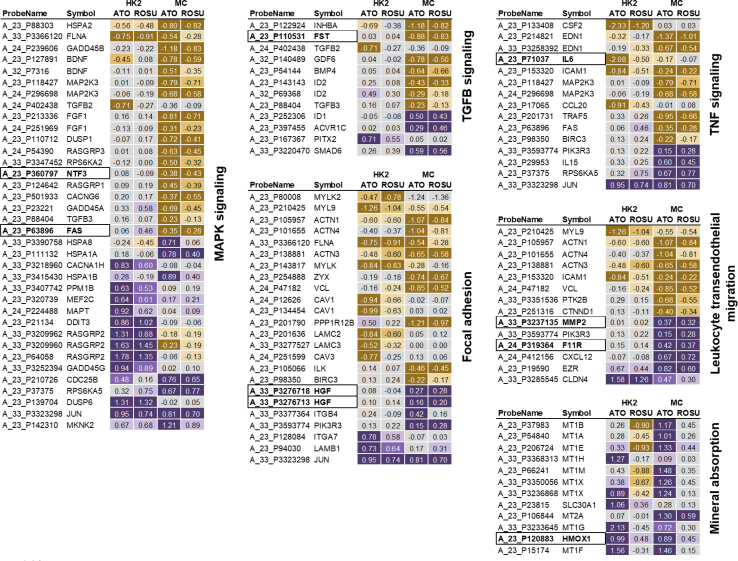
Heatmap of pathway-related genes and biomarkers Log fold-change values of DEGs assigned to enriched molecular pathways are depicted as heatmaps with purple indicating significant upregulation and yellow indicating significant downregulation after statin treatment. Selected molecular biomarkers that have been evaluated in the AURORA study are depicted in bold font with a border. (For interpretation of the references to colour in this figure legend, the reader is referred to the Web version of this article.)

The following eight biomarkers that are linked to the affected molecular processes after statin treatment in renal cells were available in patients at baseline in the AURORA study (HGF, IL6, MMP2, FAS, NTF3, HMOX1, FST, and F11R) which were subsequently evaluated regarding the effect of rosuvastatin in hemodialysis patients (see Fig. 2).

### 3.1 Biomarker-based patient phenotypes are associated with outcome in AURORA

We identified four patient phenotype clusters in the AURORA study based on the residual values of the eight measured biomarkers. The phenotype clusters ranged from a minimum of 35 to a maximum 185 patients (Table 2).

**Table 2:** Baseline characteristics of the analyzed AURORA samples and the four phenotypes.

	Overall population (n = 398)	Phenotype 1 (n = 125)	Phenotype 2 (n = 35)	Phenotype 3 (n = 53)	Phenotype 4 (n = 185)	p-value
Baseline characteristics						
Female gender (%)	83 (20.9)	28 (22.4)	10 (28.6)	14 (26.4)	31 (16.8)	0.23
Age (years)	67 [60, 74]	68 [62, 74]	69 [62, 74]	68 [59, 74]	66 [59, 73]	0.29
Years on RRT	2.7 [1.1, 6.0]	3.7 [1.7, 6.3]	1.9 [1.1, 3.8]	7.0 [1.9, 13.6]	2.1 [0.7, 4.3]	**<0.01**
Calculated K_t_/V	1.1 [1.0, 1.3]	1.1 [1.0, 1.3]	1.1 [0.9, 1.3]	1.1 [1.0, 1.2]	1.1 [1.0, 1.2]	0.75
Albumin (g/L)	39.3 (3.1)	39.6 (2.9)	37.8 (2.8)	39.0 (3.3)	39.5 (3.1)	**0.01**
Hemoglobin (g/dL)	11.8 (1.5)	11.7 (1.6)	12.2 (1.4)	11.9 (1.5)	11.7 (1.5)	0.33
hs-CRP (mg/L)	1.2 (1.2)	1.6 (1.3)	1.8 (1.4)	1.2 (1.2)	0.8 (0.9)	**<0.01**
Fasting Glucose level (mmol/L)	4.9 [4.6, 5.6]	5.0 [4.6, 5.7]	4.9 [4.3, 6.0]	4.9 [4.7, 5.4]	4.9 [4.6, 5.6]	0.83
Creatine Kinase (IU/L)	56 [36, 84]	50 [33, 82]	47 [32, 62]	49 [36, 75]	65 [41, 91]	**<0.01**
Aspartate Aminotranferase (IU/L)	16.0 (8.7)	15.6 (8.3)	15.9 (5.4)	20.2 (12.0)	15.2 (7.9)	**<0.01**
Calculated LDL-Cholesterol (mmol/L)	2.6 (0.9)	2.5 (0.8)	2.8 (1.1)	2.7 (0.9)	2.6 (0.8)	0.12
BMI (kg/m2)	25.0 (3.4)	24.8 (3.4)	24.4 (3.3)	24.5 (3.3)	25.4 (3.5)	0.2
Systolic Blood Pressure (mmHg)	138.7 (24.4)	142.8 (24.2)	129.5 (17.7)	136.2 (25.8)	138.5 (24.7)	**0.03**
Diastolic Blood Pressure (mmHg)	76.4 (12.7)	77.3 (12.9)	73.8 (12.4)	75.7 (13.3)	76.6 (12.6)	0.52
Pulse pressure (mmHg)	60 [50, 74]	69 [55, 77]	50 [43, 67]	57 [49, 75]	60 [50, 73]	**0.01**
**Medical history, n (%)**						
Current smoker	66 (16.6)	25 (20.0)	9 (25.7)	3 (5.7)	29 (15.7)	**0.05**
Diabetes	84 (21.1)	27 (21.6)	9 (25.7)	9 (17.0)	39 (21.1)	0.8
History of CVD	172 (43.2)	58 (46.4)	15 (42.9)	21 (39.6)	78 (42.2)	0.83
Peripheral artery disease	69 (17.3)	27 (21.6)	6 (17.1)	7 (13.2)	29 (15.7)	0.46
History of coronary heart disease	74 (18.6)	27 (21.6)	9 (25.7)	8 (15.1)	30 (16.2)	0.39
History of vascular disease	98 (24.6)	31 (24.8)	7 (20.0)	14 (26.4)	46 (24.9)	0.92
History of neurovascular disease	68 (17.1)	23 (18.4)	6 (17.1)	4 (7.5)	35 (18.9)	0.26
**Cause of ESRD, n (%)**						0.21
Diabetes	54 (13.6)	18 (14.4)	6 (17.1)	5 (9.4)	25 (13.5)	
Genetic conditions	51 (12.8)	16 (12.8)	5 (14.3)	2 (3.8)	28 (15.1)	
Glomerulonephritis or Vasculitis	69 (17.3)	24 (19.2)	3 (8.6)	12 (22.6)	30 (16.2)	
Nephropathy or Nephrosclerosis	94 (23.6)	32 (25.6)	5 (14.3)	9 (17.0)	48 (25.9)	
Pyelonephritis or Interstitial	63 (15.8)	19 (15.2)	9 (25.7)	13 (24.5)	22 (11.9)	
Unknown/unspecified	44 (11.1)	10 (8.0)	3 (8.6)	8 (15.1)	23 (12.4)	
Other	23 (5.8)	6 (4.8)	4 (11.4)	4 (7.5)	9 (4.9)	

Key clinical parameters of the investigated sample set of the AURORA trial are given for the whole cohort as well as for the four identified phenotypes. Mean values plus standard deviation are provided for normally distributed continuous variables with median values plus interquartile ranges for non-normally distributed continuous data. Categorical variables are expressed as frequencies (percentages). Years on RRT = years on renal replacement therapy; hs-CRP = high sensitivity C-reactive protein; BMI = body mass index; ESRD = end-stage renal disease.

The largest cluster (phenotype 4, n = 185), labelled reference phenotype, had the lowest concentration of hs-CRP (p < 0.01) and showed the highest levels of both aspartate aminotransferase (p < 0.01) as well as creatine kinase (p < 0.01). Phenotype 1 exhibited higher pulse pressure (p = 0.01), whereas phenotype 2 showed higher proportion of smokers (p = 0.05). Patients in phenotype 3 had significantly longer dialysis vintage (7.0 years as compared to 2.7 years of the overall population) (p < 0.01).

**Fig. 3:**
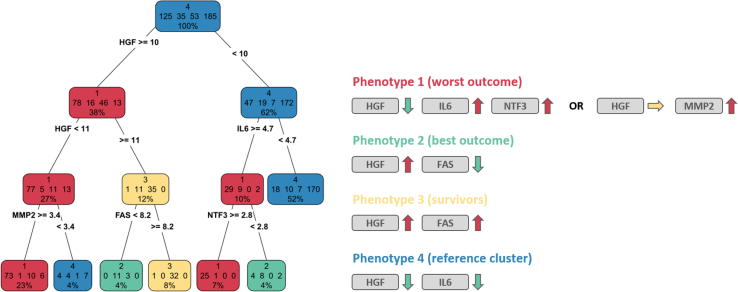
Biomarker relevance per patient phenotype A decision tree was constructed to categorize phenotypes based on biomarker levels. Color-coding of nodes in the decision tree reflect the dominant phenotype (red = phenotype 1; green = phenotype 2; yellow = phenotype 3; blue = phenotype 4). Simplified rulesets for all terminal nodes holding more than 10 patients of the dominant phenotype are given on the right. Phenotype 3 for example is characterized by high levels of HGF as well as FAS. (For interpretation of the references to colour in this figure legend, the reader is referred to the Web version of this article.)

The decision tree that was built with the eight biomarkers identified distinctive biological profiles (Fig. 3). The global accuracy of the decision tree was 81.9 %. Phenotype 1 had low HGF (a protein upregulated by statins) and high IL6/NTF3 (proteins down-regulated by statins) levels or moderately low HGF and high MMP2 levels respectively. In contrast, phenotype 2 had high HGF (a protein upregulated by statins) and low FAS (a protein down-regulated by statins) levels.

Phenotype 1 was significantly associated with increased rates of CV death, all-cause mortality, and MACE in crude models [HR = 1.78; 95 % CI (1.26–2.52), HR = 1.72; 95 % CI (1.28–2.30), and HR = 1.62; 95 % CI (1.19–2.22), respectively] as compared with the reference phenotype (Table 3). After adjustment for clinical and biological factors, phenotype 1 remained significantly associated with CV death and MACE [adjusted HR = 1.55; 95 % CI (1.03–2.34) and HR = 1.48; 95 % CI (1.03–2.14), respectively] and showed borderline association with all-cause mortality [adjusted HR = 1.35; 95 % CI (0.95–1.91) (Table 3).

**Table 3:** Association of patient phenotypes with outcome.

	Univariable		Multivariable	
	HR (95 % CI)	p-value	HR (95 % CI)	p-value
**CV death (n = 155)**				
**Per phenotype**				
Phenotype 1	1.78 (1.26–2.52)	**0.001**	1.55 (1.03–2.34)	**0.036**
Phenotype 2	0.71 (0.36–1.43)	0.34	0.74 (0.36–1.52)	0.42
Phenotype 3	0.94 (0.55–1.61)	0.82	0.83 (0.46–1.50)	0.54
Phenotype 4	–	–	–	–
**All-cause mortality (n = 230)**				
**Per phenotype**				
Phenotype 1	1.72 (1.28–2.30)	**0.0003**	1.35 (0.95–1.91)	0.09
Phenotype 2	0.86 (0.51–1.47)	0.59	0.80 (0.46–1.39)	0.44
Phenotype 3	1.18 (0.78–1.77)	0.43	1.04 (0.67–1.62)	0.86
Phenotype 4	–	–	–	–
**MACE (n = 199)**				
**Per phenotype**				
Phenotype 1	1.62 (1.19–2.22)	**0.002**	1.48 (1.03–2.14)	**0.035**
Phenotype 2	0.73 (0.40–1.33)	0.30	0.78 (0.42–1.46)	0.43
Phenotype 3	1.18 (0.77–1.83)	0.45	1.12 (0.69–1.79)	0.65
Phenotype 4	–	–	–	–

Hazard ratios of univariate and multivariate Cox proportional hazards regression models are provided for the three phenotypes with respect to the reference phenotype 4. Parameters included in the multivariate analysis for adjustment included age, albumin, log hs-CRP, history of CV disease, diabetes mellitus, dialysis vintage and log BNP.

### 3.2 Biomarker-based patient phenotypes are associated with response to statin therapy

**Fig. 4:**
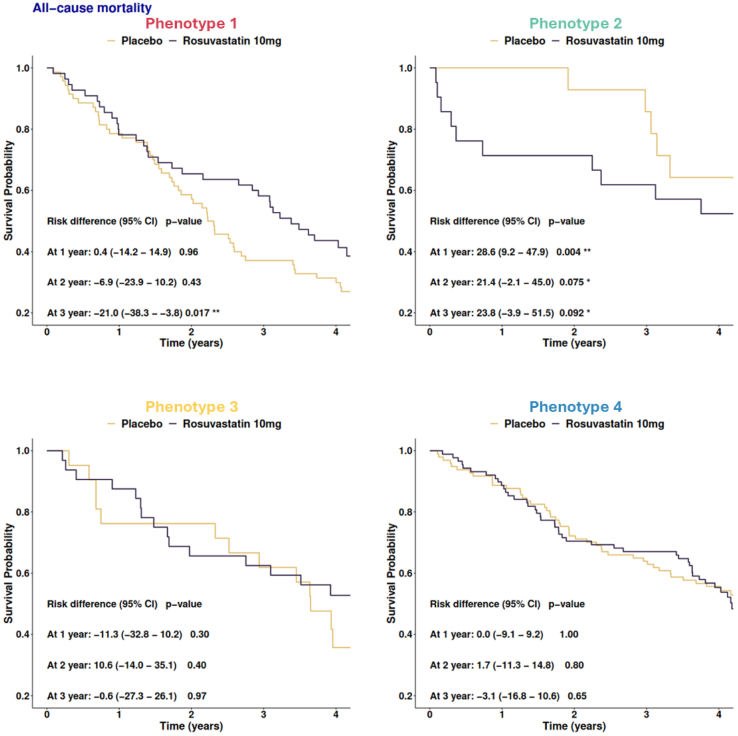
Association of treatment response with outcome Kaplan-Meier plots for the four phenotypes showing significant associations with the outcome all-cause mortality when comparing rosuvastatin treated vs untreated patients per phenotype. * *p*-value <0.1 ** p-value <0.05.

Rosuvastatin was associated with significantly lower risk of all-cause mortality at 3 years [risk difference = −21.0; 95 % CI (−38.3 to −3.8); p = 0.017] in phenotype 1. In contrast, rosuvastatin was significantly associated with increased risk of all-cause mortality at 1 year and tended to be associated with high all-cause mortality at 2 and 3 years in phenotype 2 (Fig. 4). No significant differences regarding treatment response as compared with placebo were identified for phenotypes 3 and 4 [all p > 0.5 at risk difference 1, 2, and 3 years] (Fig. 4). A similar pattern was observed when considering MACE and CV death.

## 4 Discussion

In this translational study we investigated the impact of statin treatment on renal cells in-vitro on the transcriptional levels and evaluated a set of biomarkers being linked to statin-affected molecular pathways in plasma samples of patients from the AURORA trial regarding their association with disease outcome and response to statin treatment. We show that statins had a larger impact on gene expression changes in MCs as compared to HK2 cells. Atorvastatin had a larger effect on expression changes than rosuvastatin with a number of transcripts, however, being affected in a consistent way by both statins being an indication for a strong drug class effect. Key affected molecular pathways after statin treatment in renal cells included the fibrosis-associated TGFB signaling pathway, the inflammatory processes of TNF signaling and leukocyte trans-endothelial migration, focal adhesion, mineral absorption as well as MAPK signaling. These findings are in-line with recently reported effects of statin treatment in the context of contrast-induced nephropathy [20]. When building phenotypes based on the eight selected plasma protein biomarkers in the AURORA trial, phenotype 1 was associated with worse outcome overall, but appeared to have improved outcome when treated with statins. Interestingly, these patients had low HGF levels at baseline, a marker that is up-regulated by statins, and high IL6 and NTF3 levels, two proteins that are down-regulated by statins. In contrast, phenotype 2 was characterized by high HGF and low FAS levels respectively. These patients experienced worse outcome when treated with statins as compared to untreated patients with this molecular phenotype.

Traversing finding from the in-vitro setting to the human situation is in most cases challenging and requires quality control at various steps. We first investigated the changes in gene expression of the direct statin drug target and associated proteins. Based on data from the Human Protein Atlas, there is low protein expression of the direct drug target of statins, HMGCR, in glomeruli and medium protein expression in tubular cells [21]. HMGCR was significantly upregulated in MCs in our analysis on the transcriptional level after statin treatment. This upregulation of HMGCR is most likely a cellular counter mechanism due to reduced intracellular cholesterol concentrations being in-line with reports from other groups [22,23]. In addition to HMGCR, also the receptor for low density lipoprotein (LDLR) was reported to be upregulated after statin treatment, a phenomenon that we also detected in MCs but not in HK2 cells [23]. Lower intracellular cholesterol levels not only increase expression of LDLR but also expression of the proprotein convertase subtilisin/kexin type 9 (PCSK9), another molecule with induced gene expression in MCs in our study. Squalene epoxidase (SQLE), one of the rate-limiting enzymes in the sterol biosynthesis pathway was also significantly upregulated in MCs after statin treatment in our experiments [24]. These findings solidified the conduct of the performed in-vitro gene expression experiments. Further evidence regarding the quality of the in-vitro data was found when inspecting the set of genes that were significantly regulated by both statins in both cell lines. Among the significantly downregulated molecules was the connective tissue growth factor (CTGF), a pro-fibrotic stimulus, which has been shown to be affected by statin treatment, both in renal as well as endothelial cells [[25], [26], [27]]. The Kruppel like factor 2 (KLF2) was among the significantly upregulated molecules by both drugs in both cell lines. KLF2 was reported to be downregulated after high glucose treatment in human endothelial cells and reduced KLF2 expression was associated with aggravated endothelial injury in DN [28,29].

The affected genes in the cell culture setup that were responsible for patient stratification into the four endophenotypes in the AURORA were HGF, IL6, MMP2, FAS, and NTF3. HGF is a cytokine with a number of regulatory functions in the context of immunomodulation, angiogenesis, morphogenesis, as well as tissue repair [30]. HGF was shown to be elevated in hemodialysis patients and a link to cardiovascular risk was postulated although it is still under debate whether HGF increases CVD risk or whether it is actually involved in counterbalancing endothelial damage [[30], [31], [32]]. Burgazli and colleagues reported on the role of statins to modulate HGF-induced proliferation of human umbilical vein endothelial cells [33]. Our data rather indicate that HGF has a protective role with phenotype clusters showing higher values of HGF being linked to improved outcome whereas phenotype cluster 1 with the worst prognosis showed the lowest HGF values. This cluster was also characterized by high MMP2 levels, a molecule involved in fibrotic processes. HGF was previously reported to induce MMP2 levels in human glomeruli next to reducing levels of TGFB1 and type IV collagens thus exerting its anti-fibrotic effects [34]. MMP2 levels are elevated in ESRD with hemodialysis reducing levels of MMP2 [35]. Serum levels of MMP2 are associated with mortality in hemodialysis patients [36]. An inhibitory impact of statin treatment on MMP2 levels was also reported by de Araujo Junior and colleagues as well as by Janardhanan et al. respectively [37,38]. Statins were also shown to reduce levels of pro-inflammatory IL6 in human vascular smooth muscle cells [39] and monocytes from hypercholesterolemic patients [40]. In addition statins reduced IL6 levels in patients on hemodialysis or the pre-dialysis state [[41], [42], [43]]. High IL6 levels in our analysis where characteristic for phenotype 1 holding those patients with the worst prognosis but on the other hand showing the most benefit of statin treatment.

The analysis performed in the AURORA trial strengthens the validity of our results. First, phenotype 1 (n = 125) had the most opposing biological profile (low HGF, high IL6, and NTF3) to the expected biological modifications triggered by statins. Patients of this phenotype had the worst prognosis with respect to overall survival, CV death and also risk of MACE. In addition, this profile appeared to have some benefit from statin treatment when considering all-cause mortality. This suggests that patients in whom the biomarkers are most opposed at baseline to statin actions will experience more benefit from statins. These patients have the most severely impaired biological profile on which statins can have an impact on. The only biological feature that is not the “opposite” of statin-induced biological modification is high MMP2. This may be related to the increased inflammation and oxidative stress in hemodialysis patients, as serum MMP-2 is known as a marker of inflammation which is a key mechanistic pathway in the accelerated atherosclerosis in dialysis patients. Although MMP2 concentration decreases with the initiation of hemodialysis, it does not fall to concentrations seen in patients without ESRD [36].

In contrast, phenotype 2 (n = 35), which had spontaneously high levels of HGF and low levels of FAS appeared to experience worse outcome when treated with statins (especially when considering short-term outcomes). As these patients have already high levels of HGF and low levels of FAS, it is likely that statins cannot further modify these biological systems, or could even modify these already extreme concentrations to levels that are deleterious to patients. In other words, statins cannot do good in patients that already have a very favorable profile, but statins might induce adverse events.

The two other clusters (3 and 4) had baseline biomarker profile that were not the inverse of the expected modification induced by statins: cluster 3 had already high levels of HGF, and cluster 4 had already low levels of IL6. In these profiles, no significant response to statin therapy was detected.

This study has its limitations. First, we only had biomarker data available before initiation of rosuvastatin treatment in dialysis patients of the AURORA trial. We therefore were not able to assess the direct impact of statin treatment on concentrations of those mechanistic biomarkers that we have identified in the in-vitro setting. We were nevertheless able to identify four phenotypes based on the baseline biomarker levels that showed differences in association with outcome. Although we were able to identify these phenotypes, we cannot rule out that the patient segmentation might be enhanced by adding further markers that have not been measured in the current study. In our set of DEGs there have been a few other markers of interest that might be worth investigating in future studies like e.g. the connective tissue growth factor (CTGF), the growth differentiation factor 6 (GDF6), or the C-X-C motif chemokine ligand 12 (CXCL12). We are also aware that the cohort of hemodialysis patients is a very special one and we can only speculate at that point in time how well the identified markers will perform in non-dialysis patients with increased risk to develop CVD prone to statin therapy. Most importantly, validating this biological approach in other trials would be key to ascertain external validity.

Logical next steps for future studies are therefore to (i) investigate the potential of the proposed biomarkers in identifying statin responders in patients with advanced CKD not treated with hemodialysis as well as to (ii) measure additional biomarkers being linked to statin mechanism of action in order to evaluate whether the patient clustering can be further enhanced to properly predict statin effect.

## 5 Conclusions

In this translational study we investigated the impact of statin therapy on gene expression changes in renal cells and identified dysregulated mechanisms and markers. A panel of eight markers was able to stratify hemodialysis patients into four phenotypes that showed significant differences in patient outcome. Patients with low HGF and high IL6 appeared to experience better response to statins, while patients with high HGF and low FAS appeared to have worse outcome when treated by statins. Intermediate biological phenotype (with either low IL6 or high FAS) seemed not to be affected by statin treatment.

## Ethics approval and consent to participate

The study is being conducted in accordance with the ethical principles of the Declaration of Helsinki, the International Conference of Harmonisation Good Clinical Practice guidelines and local regulatory requirements. All randomized patients provided a written informed consent.

## Consent for publication

Not applicable.

## Availability of data and materials

Microarray data are deposited in the Gene Expression Omnibus with accession number GSE208579. All other data from this study are included within the manuscript.

## Funding

This project has received funding from the 10.13039/501100010767Innovative Medicines Initiative 2 Joint Undertaking under grant agreement No 115974. The JU receives support from the European Union's Horizon 2020 research and innovation programme and EFPIA and JDRF. Any dissemination of results reflects only the author's view; the JU is not responsible for any use that may be made of the information it contains. This project has also received funding from the European Union's Horizon 2020 research and innovation programme under the 10.13039/100010665Marie Skłodowska-Curie grant agreement No 812699 (IMPROVE-PD).

## CRediT authorship contribution statement

**Johannes Leierer:** Data curation, Formal analysis, Visualization, Writing – original draft, Writing – review & editing. **Madonna Salib:** Data curation, Formal analysis, Methodology, Visualization, Writing – original draft, Writing – review & editing. **Michail Evgeniou:** Data curation, Formal analysis, Writing – review & editing. **Patrick Rossignol:** Formal analysis, Investigation, Writing – review & editing. **Ziad A. Massy:** Formal analysis, Investigation, Writing – review & editing. **Klaus Kratochwill:** Formal analysis, Investigation, Writing – review & editing. **Gert Mayer:** Conceptualization, Supervision, Writing – review & editing. **Bengt Fellström:** Formal analysis, Investigation, Writing – review & editing. **Nicolas Girerd:** Conceptualization, Formal analysis, Investigation, Supervision, Writing – review & editing. **Faiez Zannad:** Conceptualization, Formal analysis, Investigation, Supervision, Writing – review & editing. **Paul Perco:** Conceptualization, Formal analysis, Investigation, Supervision, Visualization, Writing – original draft, Writing – review & editing.

## Declaration of competing interest

The authors declare the following financial interests/personal relationships which may be considered as potential competing interests:Paul Perco reports a relationship with Delta4 GmbH that includes: employment.Klaus Kratochwill reports a relationship with Delta4 GmbH that includes: co-founder.

## List of abbreviations

CV: cardiovascular
CVD: cardiovascular disease
DEG: differentially expressed gene
ESRD: end-stage renal disease
FDR: false discovery rate
HK2: proximal tubular human kidney cell
MACE: major adverse cardiovascular event
MC: mesangial cell
